# Effects of a *Pseudomonas* Strain on the Lipid Transfer Proteins, Appoplast Barriers and Activity of Aquaporins Associated with Hydraulic Conductance of Pea Plants

**DOI:** 10.3390/membranes13020208

**Published:** 2023-02-08

**Authors:** Elena Martynenko, Tatiana Arkhipova, Guzel Akhiyarova, Guzel Sharipova, Ilshat Galin, Oksana Seldimirova, Ruslan Ivanov, Tatiana Nuzhnaya, Ekaterina Finkina, Tatiana Ovchinnikova, Guzel Kudoyarova

**Affiliations:** 1 Ufa Institute of Biology, Ufa Federal Research Centre, RAS, Prospekt Oktyabrya, 69, 450054 Ufa, Russia; 2 Institute of Bioorganic Chemistry, Russian Academy of Sciences, Miklukho-Maklaya Str., 16/10, 117997 Moscow, Russia

**Keywords:** *Pisum sativum*, lipid transfer proteins (LTPs), Casparian bands, *Pseudomonas mandelii*, suberin, aquaporins, hydraulic conductance

## Abstract

Lipid transfer proteins (LTPs) are known to be involved in suberin deposition in the Casparian bands of pea roots, thereby reinforcing apoplast barriers. Moreover, the *Pseudomonas mandelii* IB-Ki14 strain accelerated formation of the Casparian bands in wheat plants, although involvement of LTPs in the process was not studied. Here, we investigated the effects of *P. mandelii* IB-Ki14 on LTPs, formation of the Casparian bands, hydraulic conductance and activity of aquaporins (AQPs) in pea plants. RT PCR showed a 1.6-1.9-fold up-regulation of the PsLTP-coding genes and an increase in the abundance of LTP proteins in the phloem of pea roots induced by the treatment with *P. mandelii* IB-Ki14. The treatment was accompanied with increased deposition of suberin in the Casparian bands. Hydraulic conductance did not decrease in association with the bacterial treatment despite strengthening of the apoplast barriers. At the same time, the Fenton reagent, serving as an AQPs inhibitor, decreased hydraulic conductance to a greater extent in treated plants relative to the control group, indicating an increase in the AQP activity by the bacteria. We hypothesize that *P. mandelii* IB-Ki14 stimulates deposition of suberin, in the biosynthesis of which LTPs are involved, and increases aquaporin activity, which in turn prevents a decrease in hydraulic conductance due to formation of the apoplast barriers in pea roots.

## 1. Introduction

The presence of the plant-growth-promoting (PGP) bacteria in the rhizosphere stimulates plant growth and increases their productivity in both favorable and stressful conditions [1,2,3,4]. While many different mechanisms of bacterial effect on plants have been actively researched, one aspect of the PGP bacteria interactions with plants has received less attention. This aspect is bacteria-induced changes in the formation of the apoplast barriers and their effect on the water transport in plants. These barriers are located in the endodermis and exodermis, where they appear as the suberin and lignin in Casparian bands [5]. Casparian bands and suberin lamellae are considered as key selection targets for breeding drought- and salt-tolerant crops, since the apoplast barriers prevent uncontrolled transport of water and solutes into plants [6]. The apoplastic barriers also protect plants from pathogen infection [7].

Recent research has demonstrated a connection between deposition of suberin and the salt-induced increase in abundance of the lipid transfer proteins (LTPs) in pea [8]. These proteins reversibly bind phospholipids and fatty acids (including suberin precursors) within their hydrophobic cavity, thus participating in their transfer between membranes and transport both within the cell and in the apoplast [9,10]. Expression of many LTPs can be induced by multiple biotic and abiotic stressors ([11] and references therein). We recently showed that inoculation of the rhizosphere of the durum wheat with a plant-growth-promoting *Pseudomonas mandelii* strain enhanced deposition of suberin and lignin while decreasing sodium accumulation under the conditions of elevated salinity [12]. However, the effects of these bacteria on the LTP levels in pea plants have not been studied.

Increased suberin deposition in the apoplast is beneficial for plants under salt stress conditions as a mechanism that limits apoplast permeability and delivery of toxic ions with the transpiration flow. It was shown that the aliphatic suberin confers salt tolerance to *Arabidopsis* by limiting Na+ influx [13]. However, the apoplast barriers decrease hydraulic conductance. For example, a growth-limiting supply of phosphate caused significant decreases in root hydraulic conductivity, while the formation of the apoplast barriers increased [14]. Quantification of several permeability parameters in the roots showed that suberin represents a major barrier for water flow [5]. Decreased hydraulic conductance leads to stomatal closure [15], which limits gas exchange and photosynthesis [16], while a high transpiration rate correlates with increased crop yields under favorable conditions [17]. It was suggested that restriction of water flow through the apoplast pathway may be eliminated by an enhancement of the cell-to-cell pathway through the membrane water channels—aquaporins (AQPs) [18]. In accordance with this suggestion, we demonstrated that bacteria-induced strengthening of the apoplast barriers was accompanied by increased abundance of AQPs in the roots of barley plants [19]. It was of interest to find out if bacterial inoculation influences the LTP abundance and deposition of suberin and how these effects may be related to AQP activity and hydraulic conductance of pea plants. Contribution of AQPs to the hydraulic conductivity of pea plants was previously demonstrated in the experiments showing a decrease in the average water transport rate induced by an AQP inhibitor [20]. However, this effect was not associated with formation of the apoplast barriers. Given all of the above, the aim of the present research was to elucidate the possible effects of a bacterial inoculation on the LTP abundance in unstressed pea plants and its association with suberin deposition, formation of the apoplast barriers and hydraulic conductance. We aimed to test the hypothesis that a decline in hydraulic conductance due to enhanced formation of the apoplast barriers can be compensated by an increased activity of AQPs.

The *Pseudomonas mandelii* IB-Ki14 strain was chosen for inoculation of pea rhizosphere, since it had been previously effective in stimulating suberin and lignin deposition in wheat plants [12]. The effects of bacterial inoculation on the abundance of abscisic acid (ABA) were also recorded, since this hormone is known to influence both suberin deposition in pea [21] and rice [22] and AQP abundance [23,24].

Summarizing the above, the aim of this work was (1) to find out whether bacterial inoculation affects the level of expression of genes encoding LTPs and the content of the corresponding proteins in pea plants, (2) to reveal the relationship between the level of LTP and deposition of suberin, and (3) to test the hypothesis that changes in hydraulic conductance due to formation of apoplastic barriers can be compensated by changes in AQP activity.

## 2. Materials and Methods

### 2.1. Bacterial Strain and Cultural Media

Gram-negative bacteria *Pseudomonas mandelii* IB-Ki14 (All-Russian Collection of Microorganisms B-3250) from the collection of microorganisms of the Ufa Institute of Biology of the UFIC RAS (Ufa, Russia) were used for inoculation of plants. Bacteria were cultivated in Erlenmeyer flasks with King’s B medium (2% peptone, 1% glycerol, 0.15% K_2_HPO4, 0.15% MgSO_4_·7H_2_O) on a shaker Innova 40R (New Brunswick, NJ, USA) (160 rpm) for 48 h at 28 °C.

### 2.2. Plant Growth Conditions and Treatments

Plants of *Pisum sativum* (cultivar “Sacharniy 2”) were used in the experiments. Seeds of the garden pea were sterilized by placing in a solution of 96% ethanol/3% H_2_O_2_ (1:1, *v*/*v*) for 5 min and then repeatedly washed with distilled water. Seeds were soaked in the distilled water for 24 h (with aeration for improved seed germination) and then wrapped in wet filter paper to germinate in the dark at room temperature for two days.

The plants were grown in pots (five pea seedlings per pot) with sand. To ensure drainage, a layer of gravel was placed at the bottom of pots with a volume of 500 cm^3^. After installing a glass tube for gas exchange, the pots were filled with 0.55 kg of sand sterilized by calcinations to exclude the presence of undesirable bacteria and 110 mL of 10% Hoagland–Arnon solution was added. Three-day-old seedlings (root length 1.5 cm, coleoptile length 0.5 cm) were planted and inoculated with 2 mL of the bacterial suspension of *P. mandelii* IB-Ki14 per seedlings (10^8^ CFU/mL). A subset of the plants grown in sand without the introduced bacteria was used as a control.

The plants were grown at an irradiance of 400–500 μmol m^−2^ s^−1^ PAR (ZN-500 and DNAT-400 lamps), 14 h photoperiod and 24/18 °C (day/night). Sand moisture was maintained at 80% of the water-holding capacity of sand by watering the pots daily with distilled water. The amount of water required for irrigation was calculated by weighing the pots.

### 2.3. RNA Extraction and Analysis of the Abundance of LTP mRNA

RNA was extracted from roots using the TRIzol™ Reagent (Sigma, Steinheim, Germany) according to the manufacturer’s instructions. Potentially contaminated DNA was digested with DNaseI (Synthol, Moscow, Russia) and first-strand cDNA was synthesized using the M−MLV reverse transcriptase (Fermentas, Waltham, MA, USA). Oligo(dT)15 was used as a primer, and the reverse transcription reagents were incubated at 37 °C for 1 h in a total volume of 25 μL. After tenfold dilution, 2 μL of the synthesized cDNA was used for the quantitative real-time polymerase chain reaction (qPCR). Primers for the qPCR were designed based on the cDNA sequence [25] using the PrimerQuest™ tool. The primers used for quantitative analysis of Ps-LTP1, Ps-LTP2 and Ps-LTP3 are given in Table 1.

The real-time qPCR was performed using EvaGreenI reagents (Synthol, Moscow, Russia) and a QuantStudio™5 Real-Time PCR System produced by Thermo Fisher Scientific (Applied Biosystems, Waltham, MA, USA). The qPCR protocol was as follows: 95 °C for 5 min; 40 cycles of 95 °C for 15 s and at 60 °C for 20 s and 72 °C 30 s. After the final PCR cycle, a melting curve analysis was conducted to determine the specificity of the reaction (at 95 °C for 15 s, 60 °C for 1 min and 95 °C for 15 s). The efficiency of each primer pair was determined using a 10-fold cDNA dilution series to reliably determine the fold changes. The β-tubulin gene was chosen as an internal control to normalize the amount of total RNA present in each reaction (Table 1). All reactions, including the non-template control, were performed three times. To determine the relative amount of mRNA for LTP, cycle threshold (CT) values were obtained using the CFX Connect real-time PCR Detection System software tool (Applied Biosystems, Waltham, MA, USA). To normalize the expression of the target gene, the difference between the CT of the LTP gene and the CT of β-tubulin (∆CT value) was calculated. Three independent biological replicates were performed for each variant of the experiment.

### 2.4. Immunolocalization of LTPs and the ABA

For immunolocalization of LTPs and the ABA, pieces cut from the basal part of the plant roots were fixed in 0.1 M phosphate-buffered saline (PBS) pH 7.4 containing 4% N-(3-dimethylaminopropyl)-N’-ethylcarbodiimide hydrochloride (Merck, Darmstadt, Germany) for 12 h at 4 °C and then in 4% paraformaldehyde (Riedel de Haen, Seelze, Germany) and 0.1% glutaraldehyde (Sigma, Steinheim, Germany). Fixed root tissues were then washed three times with phosphate buffer and after dehydration in a series of increasing ethanol concentrations were embedded in the JB4 resin (Electron Microscopy Sciences, Hatfield, PA, USA). Histological cross-sections (slices) with a thickness of 1.5 μm were obtained on a rotary microtome (HM 325, MICROM Laborgerate, Walldorf, Germany).

After applying blocking solution for 30 min (PBS containing 0.2% gelatin and 0.05% Tween-20), root cross-sections were incubated with the polyclonal rabbit anti-LTP (1:200 dilution) or anti-ABA (1:80 dilution) sera overnight at 4 °C. The interaction of polyclonal rabbit anti-LTP antiserum with pea LTPs was previously evaluated using Western blotting and ELISA assays [25,26]. Specificity of immunostaining for ABA has been confirmed by increased staining in the plants treated with exogenous ABA (positive control) as well as by decreased staining in the case of ABA-deficient mutant (negative control) [24].

The root slices were then washed three times in PBS with 0.05% Tween-20 followed by 3 h of incubation at 37 °C with anti-rabbit IgG secondary antibodies conjugated to Alexa Fluor 555 (Invitrogen, Rockford, IL, USA). The slices were additionally rinsed five times with PBS, covered with glass and then imaged by confocal microscopy using an FV3000 Fluoview (FV31-HSD) (Olympus, Tokyo, Japan) and laser excitation line of 561 nm. Fluorescence emission was detected at 568 nm. Detection occurred in the integration frame mode for imaging with a count of 4.

### 2.5. Suberin Detection

Freehand cross-sections from the basal part of the plant roots were stained with an alcoholic solution of Sudan III (Sigma, St. Louis, MO, USA) to reveal development of the Casparian bands [27]. Suberized tissues were stained dark orange. The date of sampling was chosen on the basis of previous experiments with salt treatment of pea plants, which showed increased deposition of suberin [8,21].

### 2.6. Parameters of Water Relations

On the 7th day after the inoculation, 50 mL of Fenton’s reagent (solution of hydrogen peroxide with ferrous iron: a mixture of Fe^2+^ 0.83 g/L and 100 μL of 30% H_2_O_2_) was added to half of the pots with control pea plants and plants inoculated with *P. mandelii* to inhibit aquaporins. Fenton reaction produces highly reactive hydroxide radicals, which react with aquaporins (and other proteins), thereby disrupting their function [28]. In the plants treated with Fenton reagent, parameters of water relations were measured simultaneously with the control plants.

All measurements were taken on the same 7th day after inoculation.

To measure transpiration, pots with plants (5 pots per variant of treatment) were covered with a polyethylene film with holes for plants to prevent evaporation from the surface, and transpiration was assessed gravimetrically as pot weight loss measured every 10 min for 1 h.

When a decline in transpiration was detected in the plants treated with Fenton reagent, water potential of disks cut from the differentiated leaves was measured with a psychrometer (PSYPRO, Wescor, Logan, UT, USA). Hydraulic conductance of the water transport pathway from roots to leaves was calculated as described [21,29], using the formula: L = T/[(Ψs − Ψl)], where T is the transpiration measured during the last 10 min before sampling to measure the water potential, and Ψs and Ψl are the water potentials of the nutrient solution and leaf, respectively.

### 2.7. Statistics

The data were statistically processed using standard MS Excel programs. Figures show means and their standard errors (s.e.). The significance of differences was assessed by *t*-test or ANOVA followed by Duncan’s test (*p* ≤ 0.05).

## 3. Results

**Figure 1:**
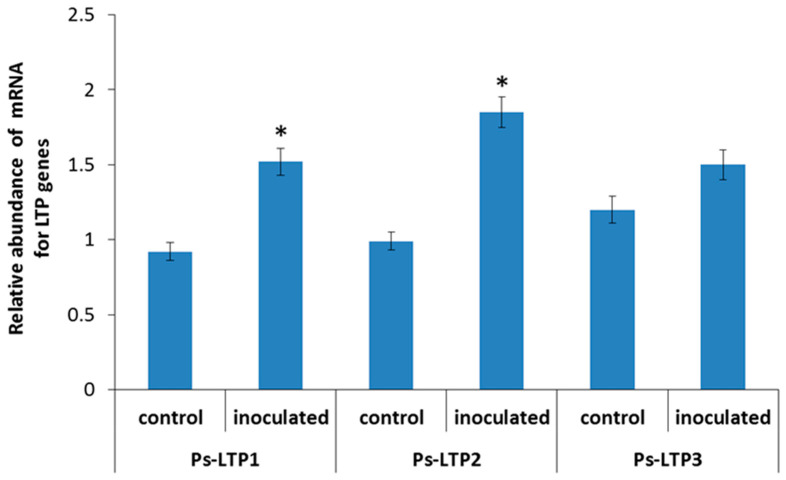
Relative abundance of transcripts of Ps-*LTP* genes (means ± s.e.) in roots of control pea plants and those inoculated with *Pseudomonas mandelii* IB-Ki14. The fold changes of genes normalized using the pea gene encoding tubulin (GenBank accession number X54844.1) are presented as relative units compared to control. Data sets marked with asterisks are significantly different from control (*n* = 3, *t*-test).

To achieve the first stated objective, we examined the effects of bacterial inoculation on the expression of the LTP encoding genes. Quantitative PCR showed that bacterial treatment significantly increased the relative amount of Ps-LTP1 (1.6-fold) and Ps-LTP2 (1.9-fold) transcripts compared to the control (Figure 1). Increase in Ps-*LTP3* transcript abundance was statistically non-significant.

**Figure 2:**
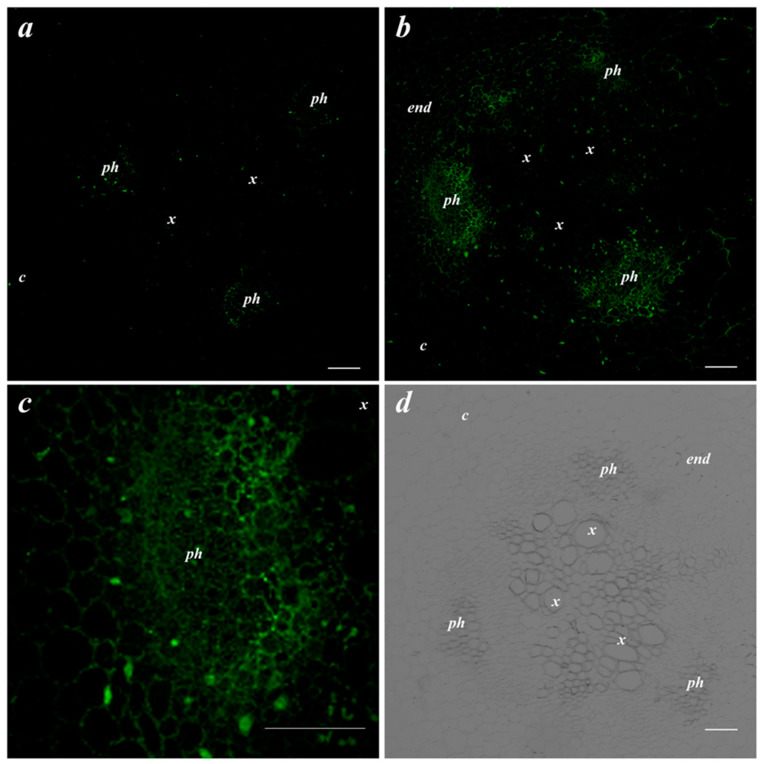
Effects of bacterial inoculation on LTP localization in the central cylinder of the basal part of the root. Root cross-section of uninoculated pea plants (**a**) and plants inoculated with *Pseudomonas mandelii* IB-Ki14 (**b**). A higher magnification image of the phloem cells from figure b (**c**). The bright field image of the central cylinder of the root (**d**). The scale bar is 50 µm. *end*—endodermis; *x*—xylem; *ph*—phloem; *c*—cortex.

Next, it was important to follow bacterial effects on LTP abundance. Immunostaining of the root cross-sections of the control pea plants revealed weak fluorescence corresponding to a low abundance of LTPs (Figure 2a). Bacterial treatment increased the brightness of fluorescence, especially in the phloem region where LTPs were present in the cell walls (Figure 2b,c).

**Figure 3:**
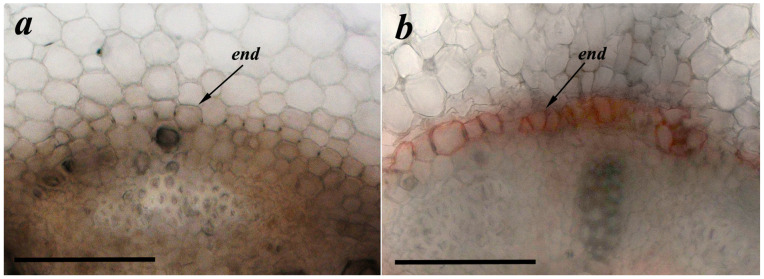
Effects of bacterial treatment on suberin deposition. Root cross-sections from basal part of pea roots untreated (**a**) and treated with *Pseudomonas mandelii* IB-Ki14 (**b**). Scale bar is 50 µm.

Effects of bacteria on deposition of suberin were studied to establish the existence of a relationship between the abundance of LTPs and formation of apoplast barriers. In contrast to control plants untreated with bacteria, suberin deposition in the endoderm region (dark orange coloration) was visible in the root sections of the plants treated with Pseudomonas mandelii IB-Ki14 (Figure 3).

**Figure 4:**
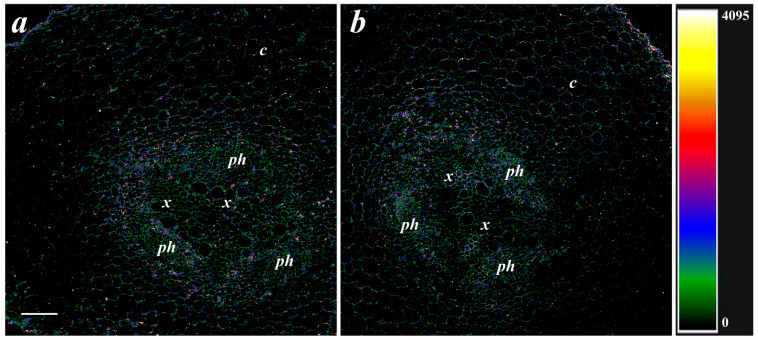
Immunolocalization of the ABA on cross-sections from basal part of pea roots untreated (**a**) and treated with *Pseudomonas mandelii* IB-Ki14 (**b**). The heatmap shows color-coded intensity of the fluorescence signal. The scale bar is 100 µm. *x*—xylem; *ph*—phloem; *c*—cortex.

Since the deposition of suberin and LTP was associated with the level of ABA in previous experiments [8], the effect of bacteria on the content of ABA in root cells was studied. ABA immunostaining with specific serum against this hormone revealed fluorescence corresponding to the presence of ABA in and around root cells. Fluorescence was brighter in the phloem and xylem located in the central root cylinder than in the cortex cells (Figure 4). There was no apparent difference between the control and treatment groups in the ABA level in the roots (Figure 4).

In further experiments, we studied the effects of bacteria-induced changes in the formation of apoplastic barriers on hydraulic conductance.

**Figure 5:**
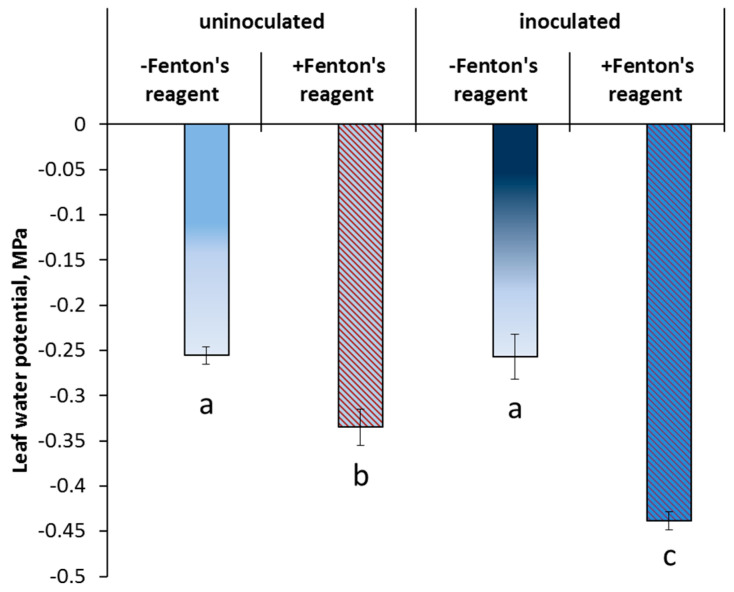
The effect of AQP inhibition on the leaf water potential (means ± s.e.) of control and inoculated with *Pseudomonas mandelii* IB-Ki14 pea plants (n = 6). Significantly different group-level means within each treatment are labeled with different letters (Duncan’s test, *p* ≤ 0.05).

The leaf water potential was not affected by bacterial inoculation (Figure 5). Inhibition of the AQP activity by Fenton’s reagent decreased leaf hydration in both treatments, although the effect was greater in the inoculated plants (70% versus 30%).

**Figure 6:**
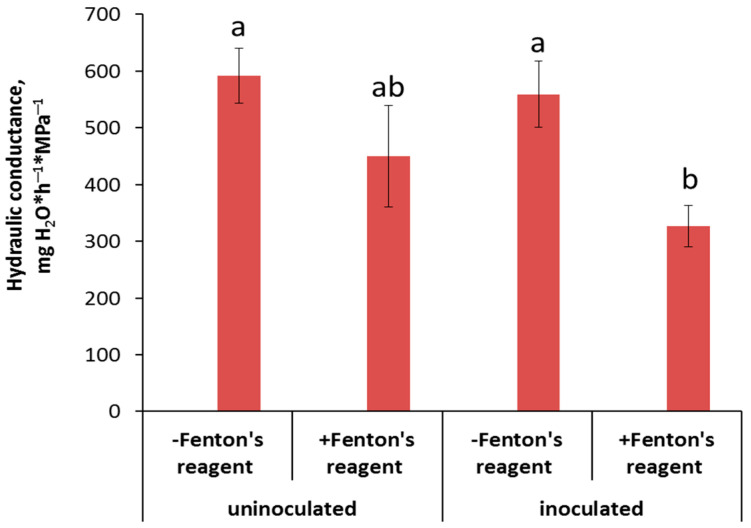
The effect of AQP inhibition on hydraulic conductance of control and inoculated with *Pseudomonas mandelii* IB-Ki14 pea plants (n = 6). Significantly different group-level means within each treatment are labeled with different letters (Duncan’s test, *p* ≤ 0.05).

Hydraulic conductance was not decreased by inoculation (Figure 6) but was lowered by inhibition of the AQP activity, although only in the inoculated plants.

## 4. Discussion

In the present research, we found that inoculation of the pea rhizosphere with *Pseudomonas mandelii* IB-Ki14 up-regulated expression of the genes encoding for LTPs, which was accompanied with an increase in the abundance of LTPs and enhanced deposition of suberin in the apoplast barriers. LTPs belong to a protein family of basic polypeptides of 9 kDa, widely distributed throughout the plant kingdom [30]. Initially, these proteins attracted the attention of researchers due to their role as pan-allergens in plant-derived food [31]. More recently, there has been a growing appreciation of the critical importance of this family of proteins in plant development and consequently research efforts focused on the unresolved questions related to their subcellular localization, expression profile and biological function [32].

One of the functions attributed to LTPs has been the intracellular lipid shuttling [33]. Our results support involvement of the pea LTPs in suberin deposition probably due to their ability to transfer hydrophobic substances including suberin precursors [10]. LTPs bind monomeric lipids in a hydrophobic pocket and transfer them through an aqueous phase [34]. LTPs have been involved in hydrophobic barrier synthesis, facilitating the transfer of precursors of lipid polymers in the apoplastic space [9]. The expression pattern of the LTP genes and extracellular localization of the proteins pointed to their role in cell wall suberization [35]. The gene encoding rice LTP2 was active in suberin-forming tissues such as roots [36]. In our previous immunohistochemical study of LTP localization, we found LTPs in the cell walls of phloem [8,21], which is in agreement with the present work. Our data suggest involvement of LTPs in the passage of suberin precursors through the hydrophilic cell wall to enable suberin deposition in the apoplast and phloem unloading. The phloem is a major pathway for assimilates and other nutrients. However, the phloem transport of lipids has been given little attention [37], since the presence of hydrophobic substances was not expected in the hydrophilic phase of sieve elements. At the same time, a discovery of lipids bound to proteins in human blood [38] raises the possibility that lipids and their respective lipid-binding proteins in the phloem may have similar functions in plants. This is supported by several reports showing the presence of LTPs and lipids in the phloem [39,40]. Proteomics of the phloem exudates showed increased content of LTPs during drought in tomato plants [41].

The effects of microorganisms on the level of LTPs in plants have been demonstrated in several reports. For instance, the *Arabidopsis* LTP3 gene transcript accumulated in response to the pathogenic *Pseudomonas* strain [42]. Transcript accumulation of the LTP4 in tobacco was increased in response to wounding and infection with *Ralstonia solanacearum* [43]. Mutation of the gene encoding for a lipid transfer protein altered cuticular lipid composition in the *Arabidopsis* and enhanced its susceptibility to infection by a fungal pathogen [44]. Moreover, LTP genes were expressed in legume roots and nodules, while RT-qPCR assays showed up-regulation of some LTP genes in *Phaseolus vulgaris* roots inoculated with rhizobia during nodulation [45]. These reports demonstrate the importance of LTPs for both pathogenic and growth-promoting effects of microorganisms, and the mechanisms of action of LTPs have been discussed in terms of their function as antimicrobial agents or their involvement in several important physiological processes in plants, including plant defense against biotic and abiotic stresses and cell signaling [46]. However, little attention was paid to the LTP involvement in suberin deposition and formation of the apoplast barriers in plants treated with bacteria. Formation of the apoplast barrier was considered as a mechanism restricting pathogens to the infection site and thus conferring pathogen resistance in plants [7]. However broader aspects of importance of the apoplast barriers in bacteria-treated plants have not been sufficiently addressed. Plant-growth-promoting bacteria have been shown to enhance deposition of lignin and suberin in salt-stressed wheat plants [12]. However, the association of bacteria-induced formation of the apoplast barriers with the bacterial effects on LTPs has been studied only in the present experiments. These experiments demonstrated that a bacteria-induced increase in deposition of suberin in the endodermis was accompanied with a corresponding increase in expression of the LTP genes and abundance of LTPs in roots.

Although enhanced formation of the apoplast barriers protects plants from unrestricted penetration of toxic ions, this effect by itself is unlikely to promote plant growth under normal conditions, since a decline in the apoplast water transport leads to stomatal closure and disturbance of the gas exchange. It was suggested [18] that a decrease in water flow through the apoplast pathway may be compensated by an enhancement of the water transport across cellular membranes through aquaporins (AQPs). The results of the present experiments with an AQP inhibitor (Fenton’s reagent) confirm this statement. We found a greater reduction in hydraulic conductance by the AQP inhibitor in inoculated plants, indicating a bacteria-induced increase in the contribution of water channels to water transport compared to control (non-inoculated) plants.

A decrease in the average water transport rate induced by an AQP inhibitor was previously demonstrated [20]. However, an association between AQP activity and formation of the apoplastic barriers has been demonstrated by us for the first time.

Previous experiments suggested that either enhanced formation of the apoplast barriers or increased AQP levels can be induced by accumulation of the ABA. This hormone influenced suberin deposition in pea [21]. ABA-induced suberin biosynthesis appeared to be a rapid although transient response in *Arabidopsis* plants [47]. ABA has been considered to play a regulatory role in potato tuber suberization [48]. This hormone was also able to influence AQP levels in barley [24]. Increased concentration of ABA up-regulated the expression of certain aquaporins to enhance drought tolerance through increased water transport in transgenic tobacco [49]. Salinity [21] and inoculation with *P. mandelii* IB-Ki14 in the present experiments increased the abundance of LTPs in pea roots accompanied with an enhanced deposition of suberin. However, although the effect of both salinity and bacterial treatment was similar in terms of their effects on LTPs and suberin levels, their mechanisms are likely to differ. Thus, the effects of salinity obviously depended on the ABA accumulation found in salt-stressed plants, while bacteria inoculation did not influence ABA content in the pea roots; therefore, bacterial effects were ABA-independent. In addition, the difference in the effect of salinity and inoculation was the absence of an effect of salinity on the expression of the LTP genes, while *P. mandelii* IB-Ki14 increased the expression of these genes. Therefore, the effects of salinity on the LTP abundance are likely to be realized at the post-transcriptional level, while the effects caused by bacteria depend on gene transcriptions.

## 5. Conclusions

It is known that the plant-growth-promoting (PGP) bacteria stimulate plant growth and development in both favorable and stressful conditions. Present research and our recent publications [12] show that one of the mechanisms of action of the PGP bacteria is an accelerated formation of the apoplastic barriers. However, the role of LTPs in bacteria-induced effects on the apoplast barriers has not been studied, although it was previously shown that LTPs participate in suberin deposition in the Casparian bands. Here, we studied the effects of one of the PGP bacteria, *Pseudomonas mandelii* IB-Ki14, using pea *Pisum sativum* as the model plant. We investigated suberin deposition, abundance of LTPs, hydraulic conductance and activity of aquaporins in the roots of inoculated and non-inoculated pea plants. Up-regulation of pea LTP-coding genes, accumulation of LTP proteins in cell walls of the phloem as well as increased suberin deposition in the Casparian bands were detected in the roots of bacteria-treated pea plants. A greater decrease in hydraulic conductance was observed in inoculated plants after treatment by an AQP inhibitor (Fenton reagent), suggesting higher activity of AQPs in the treated plants. Thus, we have shown that *P. mandelii* IB-Ki14 stimulates deposition of suberin, in the biosynthesis of which LTPs are involved, and increases aquaporin activity to compensate for a possible decrease in hydraulic conductivity due to formation of the apoplast barriers in pea roots.

The obtained results deepen our understanding of the mechanisms that control the formation of apoplastic barriers and the activity of aquaporins, which are important in controlling water uptake and plant resistance to water deficit and salt stress. We demonstrated (i) the ability of bacteria to influence the formation of apoplastic barriers through LTP abundance and the influence of LTP on suberin deposition, as well as (ii) the ability of bacteria to increase AQP activity, thereby maintaining hydraulic conductivity despite an increase in apoplast barriers. Our results show that selection of bacterial strains with the aim of improving crop productivity and drought resistance should take into account the ability of the bacteria to influence these processes.

## Figures and Tables

**Table 1:** Sequences of primers used for qRT-PCR.

Genes	Strand	5′ to 3′ Primer Sequences	GenBank Accession Number
Ps-LTP1	Forward	GCTGCCGGTTCTATTCCTAAA	KJ569141
	Reverse	GTTGGTGGAGGTACTGATCTTG	
Ps-LTP2	Forward	TGGTAGTTATTGCGCCTATGG	KJ569142
	Reverse	GGTGGAGGACTGGCATTATTAG	
Ps-LTP3	Forward	TGGCGATGTGCATGTTAGT	KJ569143
	Reverse	GGGTGAAGGACTGGCATTATTA	
β-tubulin	Forward	GCTCCCAGCAGTACAGGACTCT	X54844.1
	Reverse	TGGCATCCCACATTTGTTGA	

## Data Availability

Not applicable.
